# Altered Expression of TSPAN32 during B Cell Activation and Systemic Lupus Erythematosus

**DOI:** 10.3390/genes12060931

**Published:** 2021-06-18

**Authors:** Paolo Fagone, Katia Mangano, Roberto Di Marco, Zyanya Reyes-Castillo, José Francisco Muñoz-Valle, Ferdinando Nicoletti

**Affiliations:** 1 Department of Biomedical and Biotechnological Sciences, University of Catania, 95124 Catania, Italy; kmangano@unict.it (K.M.); ferdinic@unict.it (F.N.); 2 Department of Medicine and Health Science “V. Tiberio”, Università degli Studi del Molise, 8600 Campobasso, Italy; roberto.dimarco@unimol.it; 3 Instituto de Investigaciones en Comportamiento Alimentario y Nutrición (IICAN), Centro Universitario del Sur, Universidad de Guadalajara, Guadalajara, Jalisco 49000, Mexico; zyanya.reyes@cusur.udg.mx; 4 University Center for Health Science, Department of Molecular Biology and Genomics, University of Guadalajara, Jalisco 49000, Mexico; biologiamolecular@hotmail.com

**Keywords:** tetraspanins, TSPAN32, systemic lupus erythematosus, plasmablasts, B cells

## Abstract

Systemic lupus erythematosus (SLE) is a chronic inflammatory disease with various clinical features. Autoreactive B cells play a role in disease pathogenesis, through the production of multiple autoantibodies, which form immune complexes and induce the inflammatory response and tissue damage associated with SLE. Recently, tetraspanins, and in particular, TSPAN32, have been recognized to play a central role in immunity, as they are involved in various biological processes, such as the antigen presentation and the activation of lymphocytes. Evidence suggests that tetraspanins could represent in the future a target for therapeutic purposes in patients suffering from autoimmune/immunoinflammatory disorders. In the present study, by performing in silico analyses of high-throughput data, we evaluated the expression levels of TSPAN32 in B cell activation and investigated its modulation in circulating B cells from SLE patients. Our data show that B cell activation is associated with a significant downregulation of TSPAN32. Additionally, significantly lower levels of TSPAN32 were observed in circulating plasmablasts from SLE patients as compared to healthy donor plasmablasts. In addition, type I interferons (IFNs)-related genes were enriched among the genes negatively correlated to TSPAN32, in SLE plasmablasts. Accordingly, IFN-α is able to induce a dose-dependent downregulation of TSPAN32 in B cells. Overall, the data here presented suggest the potential use of TSPAN32 as a diagnostic marker and therapeutic target for the evaluation and management of humoral immune responses in chronic diseases, such as SLE.

## 1. Introduction

Systemic lupus erythematosus (SLE) is an autoimmune disorder characterized by a variety of clinical manifestations and by periods of stable disease and flares [1]. Unspecific systemic symptoms (such as fever, generalized malaise, and fatigue) are more frequently present at the beginning of the disease, and also during the periods of flare [1]. The most common acute SLE manifestation is a photosensitive erythema, mainly on the face (typically the cheeks, also known as the “butterfly” rash), ears, neck and chest, and extensor surfaces of the arms. Kidney involvement is present in about 40% of cases. Patients with more severe proliferative forms of glomerular damage generally have a microscopic hematuria and/or frank proteinuria. The most common central nervous system (CNS) manifestations are cognitive dysfunction, headaches, seizures, and psychosis. Finally, transient ischemic attacks are increased in patients with SLE, due to general inflammation that promotes atherosclerosis and vasculitis and possible development of anti-phospholipid antibody syndrome, which may “per se” cause thrombotic episodes [1,2].

SLE etiopathogenesis is multi-factorial, arising from gene–environment interactions that lead to loss of tolerance toward self-antigens and to the subsequent production of autoantibodies, such as anti-DNA and anti-RNA antibodies. These autoantibodies are considered the serological hallmark of SLE [1].

Loss of self-tolerance to nuclear antigens is responsible for SLE, but reinforcement of the autoimmune responses may favor the development of an end-stage organ disease, including glomerulonephritis [1]. Escape of self-reactive B cells from tolerance mechanisms, coupled to the over-activation of B cells promoted by T cells and APCs full stimulation, contributes to abnormal B cell responses, and is involved in SLE pathogenesis. Along this line, targeting B cells and B cell-related pathways is thought to be a promising strategy for the management of SLE. The efficacy of the pharmacological targeting of B cells is supported by the use of Belimumab, which inhibits the B cell-activating factor (BAFF), and of rituximab, an anti-CD20 monoclonal antibody, for the treatment of refractory SLE. Hence, advances in the knowledge of the B cell pathophysiology in SLE may favor the design of novel therapeutic avenues [3,4].

Several lines of evidence have also indicated that type I interferon-α could contribute to the pathogenesis of SLE and it is known that the expression of several IFN-regulated genes is increased in SLE patients. Additionally, SLE patients have high serum levels of IFN-α. It has been proposed that these IFN-stimulated genes contribute to a sustained inflammation in SLE patients, by acting in a positive feedback loop [5,6].

Tetraspanin 32 (TSPAN32) is a cell-membrane protein found in hematopoietic progenitors and mature cells of the lymphoid, erythroid, and myeloid lineages [7]. Previous studies by our group have investigated the role played by TSPAN32 in CD4+ T cells and in multiple sclerosis (MS) [8,9]. In particular, we demonstrated that pathogenetic T cells from MOG-induced experimental autoimmune encephalomyelitis (EAE) mice showed diminished TSPAN32 levels in comparison to irrelevant T cells, and that following activation, CD4 T cells from MS patients had significantly lower mRNA levels of TSPAN32 [8]. Additionally, lower levels of TSPAN32 expression were associated to a shorter period of stable disease [9].

However, no data are currently available on the role of TSPAN32 in the regulation of humoral immune responses. The unique property of TSPAN32 of regulating the immune responses offers the possibility to develop novel therapeutic opportunities for autoimmune disorders.

In the present study, by performing in silico analyses of high-throughput data, we determined the modulation of the expression levels of TSPAN32 in B cells following activation upon exposure to different stimuli. Next, we investigated the expression levels of TSPAN32 in circulating B cells from patients with SLE. The results of our study may set the basis for the design of novel strategies for the treatment of SLE.

## 2. Materials and Methods

### 2.1. Profiling of TSPAN32 Transcription in B Cell Activation

In order to investigate the transcription levels of TSPAN32 during the activation of B cells, publicly available datasets from the Gene Expression Omnibus (GEO; https://www.ncbi.nlm.nih.gov/gds, accessed on 2 March 2021) database were retrieved and interrogated. To this aim, we selected the GSE15606, GSE20477, GSE35998, GSE84948, and GSE147497 datasets. Briefly, the GSE15606 dataset included data from murine primary B-lymphocytes purified from spleens and stimulated with IL-4 (5 ng/mL) and CD40L (200 ng/mL) and anti-mouse IgM (2.5 μg/mL) for 3 h. Three biological replicates were included. The Illumina MouseWG-6 v2.0 expression beadchip was used for the generation of the dataset and raw signal intensities of genes were normalized by cubic spline method [10]. The GSE20477 dataset included the expression data of primary B lymphocytes stimulated with 10 μg/mL anti-IgM for 30 min, then incubated for 0.5, 1, 3, and 6 h. The data were generated using the Illumina mouseRef-8 v1.1 expression beadchip array [11]. The GSE35998 dataset contains gene expression profiles of naïve B cells, B cells activated with LPS, and B cells activated with LPS + anti-CD40 for 24 and 72 h (performed in quadruplicates). The Affymetrix MG 430 2.0 arrays were used and raw data were preprocessed using the GCRMA algorithm [12]. The GSE84948 dataset includes expression data from B cells co-cultured in the presence of either Th1- or Th2-polarized CD4^+^ T cells for 1–4 days. The Affymetrix Mouse Genome 430 2.0 Array was used and the raw signals were normalized using the MAS5 algorithm [13]. Finally, the GSE147497 dataset included data from B cells activated with LPS and collected at 3 and 10 h post-stimulation, as well as data from expanded activated B cells (defined as CD22^+^CD138^−^ cells) and plasmablasts (defined as CD22^−^CD138^+^ cells) collected at day 4 post-stimulation. Read pairs from each sample were aligned to the mouse genome and counted into 50-kB bin pairs using the R package diffHic v1.9.1. Data were normalized using the trimmed mean of M value.

### 2.2. Evaluation of TSPAN32 Levels in SLE B Cells

In order to characterize the expression levels of TSPAN32 in B cell subpopulations from SLE patients, we interrogated the GSE156751 dataset. The dataset included whole-genome expression data from 4 healthy donors and 4 SLE patients. Briefly, peripheral blood mononuclear cells (PBMCs) were collected and naive B cells (defined as CD19^+^CD27^−^CD38^−^CD43^−^CD3^−^CD14^−^CD16^−^ cells), memory B cells (CD19^+^CD27^+^CD38^−^CD43^−^CD3^−^CD14^−^CD16^−^), and plasmablasts (defined as CD19^+^CD38^+^CD43^+^CD3^−^CD14^−^CD16^−^) were isolated. The Agilent-039494 SurePrint G3 Human GE v2 8x60K Microarray 039381 was used for the generation of the dataset.

### 2.3. Gene Ontology Analysis

Gene ontology analysis was performed using the software, Metascape [14] (https://metascape.org, accessed on 30 February 2021). Metascape analysis is based on databases, including KEGG, MSigDB, and Gene Ontology. Enriched ontology terms are identified by using a hypergeometric test, and statistical significance is subjected to false discovery rate correction of the p value. Redundant groups are clustered together by calculating the pairwise similarity between enriched terms.

To deduce more biologically interpretable results, the MCODE (Molecular Complex Detection) algorithm was used to automatically identify protein within the network constructed on the selected genes.

### 2.4. Evaluation of the Effect of IFN-α on TSPAN32 Levels

To evaluate the impact of IFN-α on TSPAN32 levels, we interrogated the GSE75194 dataset [15]. For the generation of the dataset, CD19^+^ B cells isolated from splenocytes of male 6-week-old C57BL/6 mice, were treated with 5 10-scalar concentrations of IFN-α (range 0.1–1000 U/mL) for 2 h (2–3 biological replicates for each condition). The Affymetrix Mouse Gene 1.0 ST Array was used, and raw data processed using the RMA algorithm.

The levels of TSPAN32 were evaluated in splenocytes from IFN-α transgenic mice, by interrogating the GSE123549 dataset [16]. The Agilent-074809 SurePrint G3 Mouse GE v2 8x60K Microarray was used for the generation of the dataset. The raw signal intensities were log2 transformed and normalized using the quantile algorithm, as per the manufacturers’ recommendation [16].

### 2.5. Statistical Analysis

Statistical analysis for the microarray data was performed using the LIMMA (Linear Model for MicroArray) test. For RNA-Seq data, the EdgeR algorithm was used for the evaluation of the statistical significance of gene expression differences among the experimental groups. For the identification of the genes significantly correlated to TSPAN32, the cosine similarity was calculated, and the statistical significance was computed using a permutation test, with 1000 random permutations. Hierarchical clustering and gene similarity matrix were calculated using the spearman rank correlation, as similarity metrics. Unless otherwise specified, an adjusted (Benjamini–Hochberg corrected) *p*-value (adj. *p*-value, FDR: False Discovery Rate) < 0.05 was considered as the threshold for statistical significance.

## 3. Results

### 3.1. Modulation of TSPAN32 Expression in B Cell Activation

**Figure 1:**
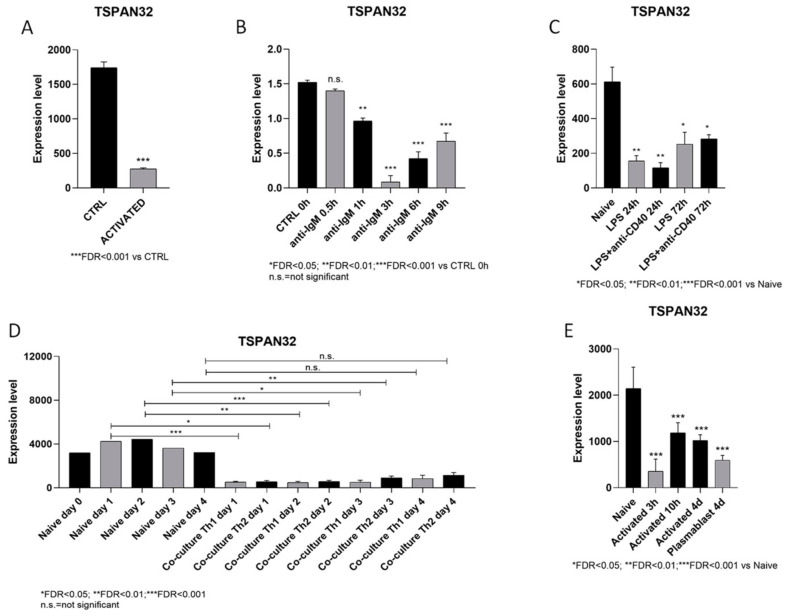
Modulation of TSPAN32 expression in B cells following activation. The transcription levels of TSPAN32 during the activation of B cells were evaluated using the publicly available GSE15606, GSE20477, GSE35998, GSE84948, and GSE147497 datasets. (**A**) TSPAN32 expression levels in murine primary B-lymphocytes purified from spleens and stimulated with IL-4 (5 ng/mL) and CD40L (200 ng/mL) and anti-mouse IgM (2.5 μg/mL) for 3 h were obtained from the GSE15606 dataset. (**B**) TSPAN32 expression data in primary B lymphocytes stimulated with 10 μg/mL anti-IgM for 30 min, then incubated for 0.5, 1, 3, and 6 h were retrieved from the GSE20477 dataset. (**C**) TSPAN32 gene expression profiles of naïve B cells, B cells activated with LPS, and B cells activated with LPS + anti-CD40 for 24 and 72 h were obtained from the GSE35998 dataset. (**D**) TSPAN32 levels in B cells cultured in the presence of either Th1 or Th2 polarized CD4^+^ T cells for 1–4 days were retrieved from the GSE84948 dataset. (**E**) TSPAN32 expression data in B cells activated with LPS and collected at 3 and 10 h post-stimulation, as well as data from expanded activated B cells and plasmablasts collected at day 4 post-stimulation were retrieved from the GSE147497 dataset.

First, we wanted to determine whether TSPAN32 is modulated following activation of B lymphocytes upon exposure to different stimuli. Figure 1A shows a significant downregulation of TSPAN32 in B cells treated for 3 h with IL-4, CD40L, and anti-IgM. Interestingly, anti-IgM treatment alone was sufficient to significantly reduce the TSPAN32 transcriptional levels (Figure 1C). Notably the effect of anti-IgM was already significant at the 1-h time-point and reached the maximum significance at 3 h (Figure 1C). Moreover, a sustained reduction of TSPAN32 was observed when either the TLR4 pathway or both the TLR4- and CD40-CD40L pathways were activated (Figure 1C). Indeed, a 3-fold reduction in TSPAN32 expression was observed at the 24-h time-point, upon LPS exposure (Figure 1C).

Next, we wanted to ascertain whether the co-culture of B cells with either Th1- or Th2-polarized T helper cells could induce a modulation of TSPAN32 expression. As shown in Figure 1D, both Th1 and Th2 cells were able to determine a significant and sustained reduction of TSPAN32 in B cells. Finally, Figure 1E shows that plasmablasts (defined as CD22^−^CD138^+^ B cells) also had significantly lower levels of TSPAN32 as compared to unstimulated naïve B lymphocytes.

### 3.2. TSPAN32 Expression Levels in SLE B Cell

**Figure 2:**
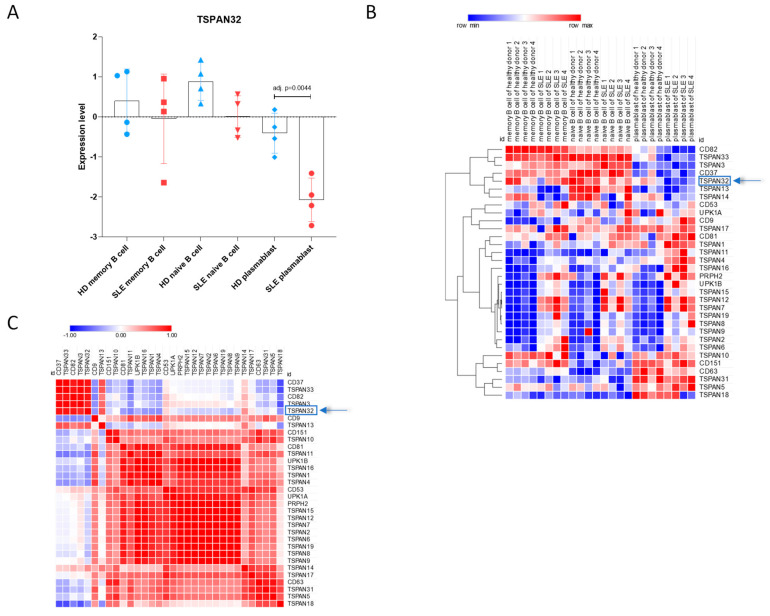
Transcriptional levels of TSPAN32 in B cell subpopulations from SLE patients. The expression levels of TSPAN32 and of the related family of tetraspanins in B cell subpopulations from 4 SLE patients and 4 healthy donors were obtained from the GSE156751 dataset. (**A**) TSPAN32 levels in naive B cells, memory B cells, and plasmablasts from SLE patients and healthy donors. (**B**) Heatmap showing the expression levels of TSPAN32 and related tetraspanins in naïve B cells, memory B cells, and plasmablasts from SLE patients and healthy donors. (**C**) Gene similarity matrix for all the analyzed tetraspanins calculated using the spearman rank correlation, as similarity metrics, on the samples included in the GSE156751 dataset.

Next, we wanted to investigate the mRNA levels of TSPAN32 in B cells from SLE patients. To this aim, we interrogated the GSE156751 dataset, which included naïve B cells, memory B cells, and plasmablasts from SLE patients, as compared to the corresponding cells from healthy people. As shown in Figure 2A, significantly reduced levels of TSPAN32 were observed in SLE plasmablasts (FDR = 0.0044). Only a trend of reduction was observed for naïve B cells (Figure 2A; FDR = 0.0543). Interestingly, only CD37, TSPAN3, CD82, and TSPAN33 showed a trend of modulation similar to that of TSPAN32 (Figure 2B,C).

**Figure 3:**
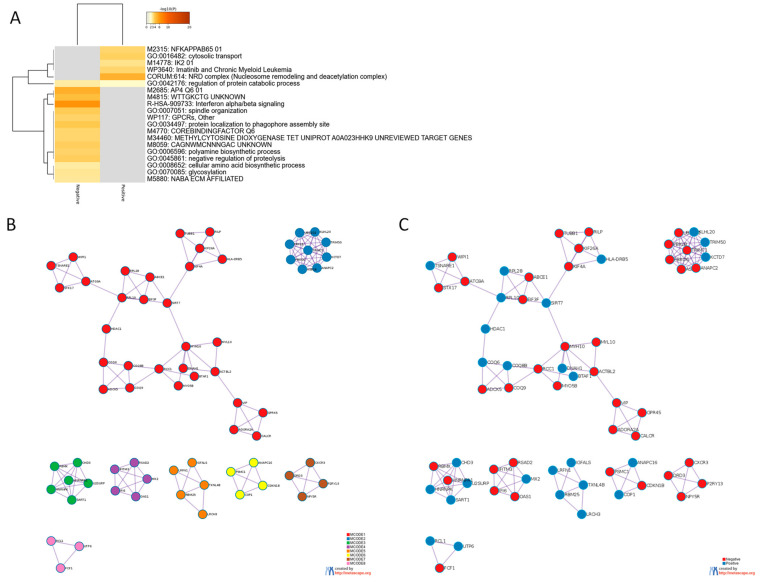
Gene Ontology and MCODE analysis for the genes that correlated most with TSPAN32 in SLE plasmablasts. Genes significantly correlated to TSPAN32 in SLE plasmablasts were identified by calculating the cosine similarity and the statistical significance was computed using a permutation test, with 1000 random permutations. (**A**) Hierarchical clustering showing the most enriched biological processes and gene ontologies among the genes significantly correlated to TSPAN32, as determined using the web-based utility, Metascape. (**B**) MCODE (Molecular Complex Detection) clusters enriched among the genes significantly correlated to TSPAN32. (**C**) MCODE clusters showing the genes based on their correlation to TSPAN32.

Next, to obtain insights on the biological meaning of TSPAN32 modulation in SLE plasmablasts, we first identified the genes significantly correlated, both positively and negatively, to TSPAN32 expression and Gene Ontology and Pathway analysis was performed. In total, 280 genes were negatively correlated while 210 genes were positively correlated to TSPAN32 (FDR < 0.1). Several enriched terms were found for both the positively and negatively correlated genes (Figure 3A). MCODE clustering analysis revealed a significant enrichment for terms related to antigen processing and presentation (MCODE1-2), spliceosome (MCODE3), Interferon α/β signaling (MCODE4), cell cycle (MCODE6), G protein signaling (MCODE7), and ribosome biogenesis (MCODE8) (Figure 3B,C; Table 1).

**Figure 4:**
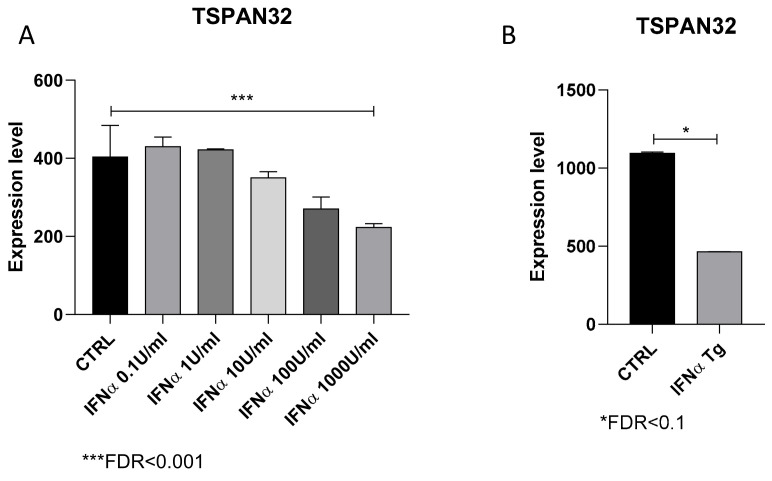
IFN-α downregulates TSPAN32 expression. (**A**) Expression levels of TSPAN32 in CD19^+^ B cells isolated from splenocytes of 6-week-old male C57BL/6mice, treated with 5 10-scalar concentrations of IFN-α (range 0.1–1000 U/mL) for 2 h, as determined from the GSE75194 dataset. (**B**) The levels of TSPAN32 were evaluated in splenocytes from IFN-α transgenic mice, as obtained from the analysis of the publicly available GSE123549 dataset.

Based on the observation of the negative correlation between TSPAN32 expression and genes belonging to the type I interferon signaling, we investigated the effect of IFN-α on TSPAN32 levels. As shown in Figure 4A, treatment of B cells with an increasing concentration of IFN-α determined a dose-dependent reduction in TSPAN32 levels, at the 2-h time-point (Figure 4A). Accordingly, TSPAN32 levels were reduced in splenocytes from IFN-α transgenic mice, who spontaneously developed an SLE-like phenotype (Figure 4B).

## 4. Discussion

Several members of the tetraspanins family are known to be implicated in the shaping of the immune responses. Notably, the tetraspanin CD81 takes part in the immune synapse, and provides a link between APCs and T cells, whereas CD37 and CD151 controls the activation of the costimulatory signaling pathways [17]. A growing body of evidence suggests that TSPAN32 functions as a negative regulator of T helper cell immune responses. Indeed, TSPAN32 ^gt/gt^ mice show augmented T cell proliferation and cytokine production [18]. We have previously shown that T cells express a steady-state level of TSPAN32, which decreases following TCR engagement. More interestingly, pathogenetic T cells from EAE mice express lower transcriptional levels of TSPAN32 and, following stimulation, T helper cells from MS patients have significantly lower mRNA levels of TSPAN32 than cells from healthy controls [8].

In the present study, by using a bioinformatic approach, we wanted to shed light on the potential role of TSPAN32 in B cells and, more specifically, in SLE. The use of whole-genome profiling data has been used lately to identify new pathogenetic avenues and therapeutic targets in a variety of settings, e.g., autoimmunity, neurodegenerative disease, and cancer [19,20,21,22,23].

We show here, for the first time, that TSPAN32 is significantly downregulated in B cells upon activation. Notably, either BCR engagement, using anti-IgM antibodies, or TLR-4 activation, via LPS treatment, are sufficient to induce a significant reduction in TSPAN32 expression. Additionally, the activation of B cells is accompanied by a sustained downregulation of TSPAN32, as significantly lower levels of this tetraspanin can be observed for at least 3 days after stimulation. We also observed that TSPAN32 undergoes significant downregulation when B cells are under the stimulation of T helper cells, including both the Th1- and Th2-polarized phenotypes. This is of interest in view of the notion that both Th1 and Th2 cytokines are deregulated in SLE patients and may contribute to the pathogenesis of SLE [24]. On a final note, we observed that BAFF stimulation is able to determine a moderate but significant downregulation of TSPAN32 expression levels (Supplementary Materials Figure S2). BAFF is a member of the tumor necrosis factor superfamily, which also includes APRIL (a proliferation-inducing ligand), CD40 ligand (CD40L), and TWEAK (TNF-related weak inducer of apoptosis). BAFF is involved during the development of B cells from the transitional maturation stage and regulates their activation, as well as the class-switch recombination process [25]. Although our data on the effect of BAFF comes only from murine B cells and primary human B cell chronic lymphocytic leukemia samples, nevertheless, we can observe a consistent reduction of TSPAN32 expression upon BAFF exposure. It is, however, apparent that the effect of BAFF is less marked than the effect achieved by anti-IgM stimulation. It will be interesting to investigate whether Belimumab treatment is able to upregulate TSPAN32 expression in pathogenetic B cells from SLE patients.

SLE is a systemic autoimmune disorder characterized by autoantibodies targeting a variety of nuclear self-antigens. These autoantibodies activate immune pathways that lead to inflammation and organ damage, in particular the kidneys [1]. Beside an altered tolerance responsible for the sustained survival of pathogenetic B cells in SLE, aberrant responses of B cells have been reported [1]. Along the same lines, co-stimulatory molecules are overexpressed on SLE T and B lymphocytes. However, the biological processes regulating B cell activation, including both the T cell-dependent and -independent pathways, remain largely unknown.

Previous data suggested that recently generated HLA-DR^high^ plasmablasts predominate over circulating HLA-DR^low^ plasma cells from SLE patients and that it is dependent on disease activity (Jacobi, 2010). Nevertheless, it is unclear whether these cells undergo maturation into plasma cells or replace the existent plasma cells in patients. Although both subsets of cells produce anti-dsDNA IgG antibodies, it is apparent that enhanced production is observed from HLA-DR^high^ plasmablasts, which underlies their pathogenic role in disease activity. Hence, expanded HLA-DR^high^ plasmablasts were proposed as a marker of B cell activation in SLE, closely related to disease activity and to the production of IgG anti-dsDNA antibodies [26]. In the present study, we expanded the observation from Jacobi et al. [26] and showed that transcriptional differences, related to TSPAN32, characterize this subset of B cells in SLE patents. We may speculate that the alteration of the control provided by TSPAN32 could represent a possible promoter of the inflammatory damage occurring in immunoinflammatory diseases, such as SLE, and hence, a newly discovered driver of autoimmunity. Here, for the first time, we propose a role for TSPAN32 in B cells and its potential role in SLE. These data set the basis for the design of new therapies for immunoinflammatory and autoimmune disorders. Interestingly, gene ontology analysis revealed a significant enrichment for processes related to spindle assembly and mitotic division among the genes negatively correlated to TSPAN32 (Supplementary Materials Table S1). On the other hand, migratory/locomotor processes are enriched among the genes positively correlated to TSPAN32 (Supplementary Materials Table S2). We could speculate that TSPAN32 might be more closely related to the activation and proliferative properties of B cells rather than on their homing and infiltrative capacities.

On a side note, we also observed significantly lower levels of TSPAN32 in circulating CD4^+^ T cells from SLE patients (Figure S1). Although, all the patients included in the analysis were not drug-naïve; hence, we cannot rule out the possibility that the reduction in TSPAN32 may be associated with the pharmacological treatment. However, this observation is in line with our previous data generated for T helper cells from multiple sclerosis (MS) patients, who were instead drug-naïve at the time of sampling [8,9].

It should be noted that Tarrant and colleagues [18] did not observe an altered activation threshold of B cells in TSPAN32 ^gt/gt^ mice. Although this seems to contrast with our observations, we may envisage the establishment of compensatory mechanisms occurring during B cell development, which may not take place in T cells. In the present study, no functional studies were performed to ascertain whether the reduced levels of TSPAN32 regulate the activation of B cells or whether the reduction of TSPAN32 is an epiphenomenon. In the latter case, the data here presented would only support the potential use of TSPAN32 as a marker of B cell activation. In this regard, it is important to point out that TSPAN32 expression in plasmablasts was found to negatively correlate with genes involved in antigen processing and presentation, cell cycle, and type I interferon signaling. Accordingly, it has been shown that anti-DNA autoreactivity is driven by type I IFN-α secretion, TLR7/9 receptor signaling, and CD40–CD40L interaction in Dnase 1l3 −/− mice [27]. Moreover, Guthridge and colleagues have shown that in SLE patients, SLEDAI scores correlated with circulating IFN-α, IP10, and IL-1α levels [28]. The recruitment of larger cohorts of subjects will be helpful to establish the modulation of TSPAN32 expression in circulating cells from patients following changes in SLEDAI score, therapeutic treatments, hormonal status, and age.

Our data also show a negative correlation between TSPAN32 expression levels and IFN-α signaling. Interestingly, expression analysis showed that IFN-α is able to significantly reduce TSPAN32 levels in B cells in vitro and IFN-α Tg mice have significantly lower levels of TSPAN32 in splenocytes. IFN-α has been proposed as a key player for SLE development. Conditional upregulation of IFN-α in mice induced typical SLE-like manifestations, such as serum immune complex, anti-dsDNA antibodies, glomerulonephritis, and alopecia. In the spleen of IFN-α Tg mice, activated effector CD4^+^ and CD8^+^ T cells, as well as B220^+^CD86^+^ B cells and CD11c^+^CD86^+^ dendritic cells were increased [16]. Accordingly, IFN-α is upregulated in the serum and in the cerebrospinal fluid of SLE patients and the levels of IFN-α have been shown to increase according to disease activity in SLE patients (reviewed in [5]). In agreement with these observations, and supported by preclinical observations that specific IFN-α inhibitors improve murine SLE [29], the development of tailored therapeutic approaches aimed at specifically inhibiting the function of IFN-α with monoclonal antibodies, anti-IFN-α receptor antibodies, IFN-α-kinoid, or downstream small molecules, targeting the Janus kinase (JAK)-signal transducer and activator of transcription (STAT) pathways, are being considered in clinical trials for SLE patients [5].

However, our study does not provide evidence of whether the reduction of TSPAN32 induced by IFN-α treatment may be a causative factor in SLE pathogenesis.

Interestingly, reduced levels of TSPAN32 were also observed in naïve B cells from SLE patients as compared to those from healthy donors. This seems to suggest that TSPAN32 may represent a sensor, or even a mediator, of the reduced threshold of activation of B cells. Future studies will be performed to assess whether the overexpression of TSPAN32 may affect B cell biology and to induce an immune tolerance state.

## Figures and Tables

**Table 1:** Gene Ontology and Pathway analysis for the MCODE subnetworks constructed on the most correlated genes to TSPAN32 in SLE plasmablasts.

Network	Annotation
MCODE_1	GO:0034497|protein localization to phagophore assembly site|-6.7;R-HSA-2132295|MHC class II antigen presentation|-6.7;GO:1901663|quinone biosynthetic process|-6.1
MCODE_2	R-HSA-983168|Antigen processing: Ubiquitination & Proteasome degradation|-17.7;R-HSA-983169|Class I MHC mediated antigen processing & presentation|-17.0;R-HSA-1280218|Adaptive Immune System|-14.2
MCODE_3	ko03040|Spliceosome|-10.9;hsa03040|Spliceosome|-10.9;R-HSA-72163|mRNA Splicing—Major Pathway|-10.2
MCODE_4	R-HSA-909733|Interferon α/β signaling|-13.1;GO:0060337|type I interferon signaling pathway|-12.4;GO:0071357|cellular response to type I interferon|-12.4
MCODE_6	R-HSA-69620|Cell Cycle Checkpoints|-7.9;R-HSA-69580|p53-Dependent G1/S DNA damage checkpoint|-7.3;R-HSA-69563|p53-Dependent G1 DNA Damage Response|-7.3
MCODE_7	WP455|GPCRs, Class A Rhodopsin-like|-8.2;R-HSA-373076|Class A/1 (Rhodopsin-like receptors)|-7.7;R-HSA-418594|G α (i) signalling events|-7.4
MCODE_8	R-HSA-6790901|rRNA modification in the nucleus and cytosol|-8.0;ko03008|Ribosome biogenesis in eukaryotes|-7.3;hsa03008|Ribosome biogenesis in eukaryotes|-7.2

## Data Availability

All data are available from Gene Expression Omnibus (GEO; https://www.ncbi.nlm.nih.gov/gds, accessed on 2 March 2021) database under accession numbers: GSE15606, GSE20477, GSE35998, GSE84948, GSE147497, GSE156751 and GSE75194.

## Supplementary Materials

The following are available online at https://www.mdpi.com/article/10.3390/genes12060931/s1, Figure S1: TSPAN32 expression in SLE CD4+ T cells. Figure S2: Modulation of TSPAN32 by BAFF; Table S1: Enrichment of pathways and biological processes among the genes positively correlated to TSPAN32 expression; Table S2: Enrichment of pathways and biological processes among the genes negatively correlated to TSPAN32 expression.
