# Supplementary material for: Altered Expression of TSPAN32 during B Cell Activation and Systemic Lupus Erythematosus
Source: Genes (Basel). 2021 Jun 18;12(6):931. doi: 10.3390/genes12060931 (PMC8234828; doi:10.3390/genes12060931)
Supplement: Supplementary file 1 [file genes-12-00931-s001.zip › suppl fig legend.pdf]

Supplementary figure legend

Figure S1. TSPAN32 expression in SLE CD4+ T cells. For the evaluation of the TSPAN32 levels in CD4+T cell from Systemic Lupus Erythematosus (SLE) patients, the GSE103760 dataset was interrogated (PMID: 33007439; DOI: 10.1016/j.clim.2020.108602). The dataset included expression levels of 8 healthy control patients (HC) and 7 SLE patients. All patients and HC donors were female, of age=33.1 (range: 24-54) and 29 (range: 27-59), respectively. All SLE patients were under pharmacological treatment (PMID: 33007439; DOI: 10.1016/j.clim.2020.108602). The Affymetrix Human Transcriptome Array 2.0 was used for the generation of the dataset. Raw data have been normalized with the RMA procedure with quantile normalization and median polish. The statistical analysis has been carried out using the Linear Model for Microarray (LIMMA) algorithm.

Figure S2. Modulation of TSPAN32 by BAFF. In order to determine the modulation of TSPAN32 expression following BAFF stimulation, the GSE54588 and the GSE89428 datasets were interrogated. GSE54588 included RNA-Seq data of murine primary B cells stimulated with anti-IgM alone, BAFF alone, or co-stimulated with both anti-IgM and BAFF for 8 and 30 hours (Almaden et al., 2014; PMID: 25497099) (A); the GSE89428 dataset included transcriptomic data from 3 primary B-cell chronic lymphoblastic leukemia before and after stimulation with BAFF for 24h (Paiva et al., 2017; PMID: 28838991) (B). The statistical analysis has been carried out using the Linear Model for Microarray (LIMMA) algorithm.
