# Supplementary figures and images for: Altered Expression of TSPAN32 during B Cell Activation and Systemic Lupus Erythematosus
Source: Genes (Basel). 2021 Jun 18;12(6):931. doi: 10.3390/genes12060931 (PMC8234828; doi:10.3390/genes12060931)

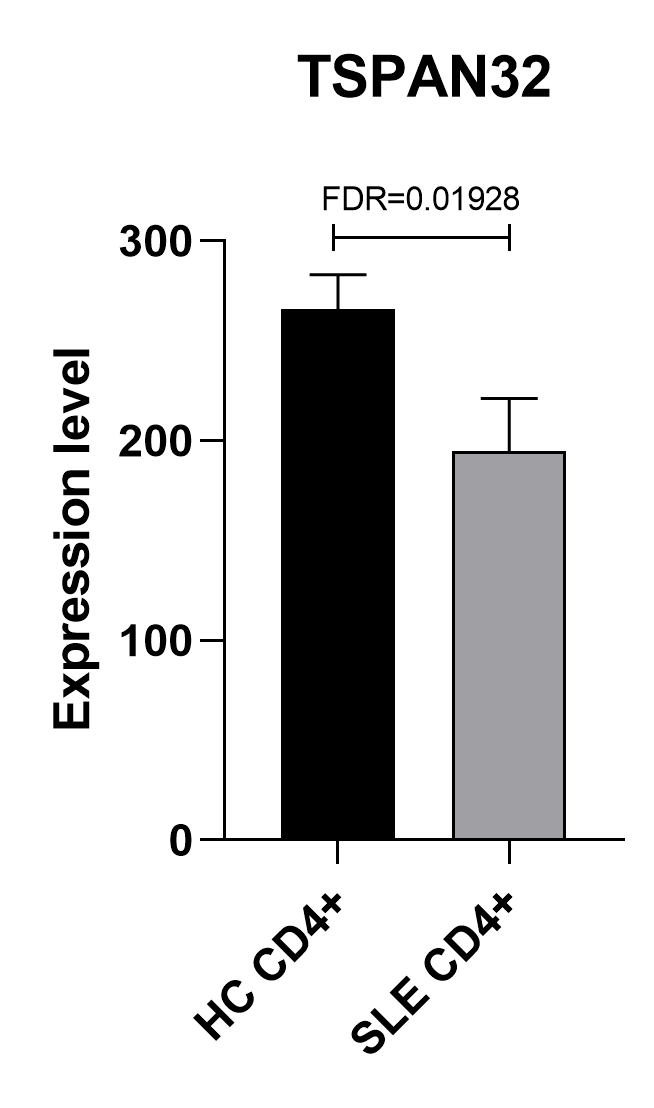

Supplement: Supplementary file 1 [file genes-12-00931-s001.zip › Suppl Figure 1.tif]

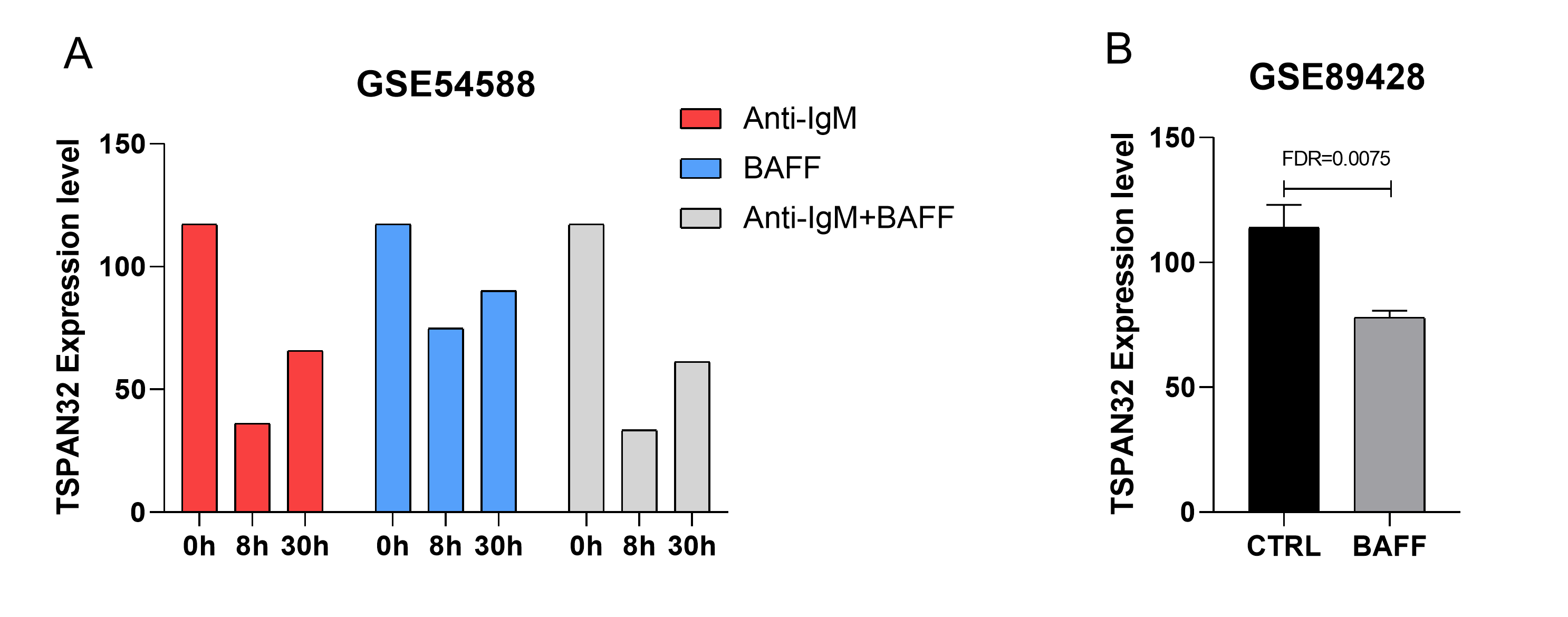

Supplement: Supplementary file 1 [file genes-12-00931-s001.zip › Suppl. Figure 2.png]
